# Supplementary material for: The genome sequence of the Poplar Grey moth, Subacronicta megacephala (Denis & Schiffermüller, 1775)
Source: Wellcome Open Res. 2024 Nov 27;9:696. [Version 1] doi: 10.12688/wellcomeopenres.23371.1 (PMC11736114; doi:10.12688/wellcomeopenres.23371.1)
Supplement: Peer review history

# Reviewer report: Reviewer response for version 1

**DOI:** 10.21956/wellcomeopenres.25766.r116100

**Published:** 2025-01-15

**Authors:** Jeffrey Marcus

**Affiliations:** 1 University of Manitoba, Winnipeg, Manitoba, Canada

In this manuscript, the authors describe the sequencing and assembly of the *Subacronicta megacephala *genome using DNA from two adult male specimens collected in the UK. The primary genome sequence assembly includes proposed chromosomal pseudomolecule sequences for 31 autosomes, the Z sex chromosome, and a complete mitochondrial genome. Gene annotation of the assembly identified 18,189 putative protein-coding genes. On the whole, this is a useful contribution to the scientific literature, but please see my comments below:

 Some suggestions to the authors:

1. Method of Specimen identification: The individual who did the specimen identification was named, but keys/species descriptions consulted, or the morphological characters used for the identification have not been included in the manuscript, unless the morphological details included in the background section were used for this purpose (it’s not clear from the text).  The text does describe that the species identification was confirmed by DNA barcoding and comparison with sequences from other specimens, which is good, but details of the method of morphological identification are still necessary.  I have made similar suggestions in previous reviews for this journal (refs 1-4). I feel strongly that such details of how morphological identifications were conducted should be a requirement for publication.
2. For future work, I suggest that the researchers preferentially sequence the heterogametic sex when assembling genomes for previously unstudied species (in the case of Lepidoptera, the heterogametic sex is female), so that draft sequence assembles can be prepared for both sex chromosomes.

Are sufficient details of methods and materials provided to allow replication by others?

Partly

Is the rationale for creating the dataset(s) clearly described?

Yes

Are the datasets clearly presented in a useable and accessible format?

Yes

Are the protocols appropriate and is the work technically sound?

Yes

Reviewer Expertise:

Evolutionary biology of insects, phylogenomics.

I confirm that I have read this submission and believe that I have an appropriate level of expertise to confirm that it is of an acceptable scientific standard, however I have significant reservations, as outlined above.

## References

[1] Marcus J. 2024. Peer Review Report For: The genome sequence of a tortricid moth, Lathronympha strigana (Fabricius, 1775) [version 1; peer review: 1 approved, 1 approved with reservations]. Wellcome Open Res 2023, 8:335. 10.21956/wellcomeopenres.21965.r96632 10.21956/wellcomeopenres.21965.r96632

[2] Kopchak, N. and J. M. Marcus 2004. Peer Review Report For: The genome sequence of the White-barred Knot-horn, Elegia similella (Zincken, 1818). [version 1; peer review: 1 approved with reservations]. Wellcome Open Res 2024, 9:186. 10.21956/wellcomeopenres.23460.r100551 10.21956/wellcomeopenres.23460.r100551 PMC1153164139494198

[3] Marcus J.M. 2024. Peer Review Report For: The genome sequence of the bramble shoot moth, Notocelia uddmanniana (Linnaeus, 1758) [version 1; peer review: 1 approved with reservations]. Wellcome Open Res 2021, 6:348. 10.21956/wellcomeopenres.19338.r79177 10.21956/wellcomeopenres.19338.r79177 PMC1133958939175988

[4] Marcus J.M. 2024. Peer Review Report For: The genome sequence of the March moth, Alsophila aescularia (Denis & Schiffermüller) [version 1; peer review: 2 approved with reservations]. Wellcome Open Res 2024, 9:50. 10.21956/wellcomeopenres.22854.r86299 10.21956/wellcomeopenres.22854.r86299 PMC1259285441210100

# Reviewer report: Reviewer response for version 1

**DOI:** 10.21956/wellcomeopenres.25766.r116103

**Published:** 2025-01-06

**Authors:** Toni de-Dios

**Affiliations:** 1 University of Tartu, Tartu, Estonia

In this piece of work, Boyes and colleagues present the first genome assembly of the Poplar Grey moth, a moth species common in the British Isles. The authors manage to assemble most of the assembled sequences into 30 autosomes, a sexual Z chromosome and a mitochondrial genome. They have also annotated the genome. Results are strongly supported by different quality control metrics. Resultant datasets and figures are available online. The methodology is adequate, and it is precisely described. The generation of this assembled genome is a step forward towards the capture of life diversity in the British Isles. In particular for this species, and as mentioned by the authors, it could help to get a better understanding of its phylogenetic relationships with other moths and population genetics.

 My only minor observation is that the first sentence is a bit difficult to read and might need to be rephrased. Also, in the last sentence of the second paragraph of the section

**Background**, I would refrain from using the adjective *“peculiar”*, or if they think is necessary, to explain why is so.

Are sufficient details of methods and materials provided to allow replication by others?

Yes

Is the rationale for creating the dataset(s) clearly described?

Yes

Are the datasets clearly presented in a useable and accessible format?

Yes

Are the protocols appropriate and is the work technically sound?

Yes

Reviewer Expertise:

Bioinformatics, Ancient DNA, Population Genetics, Phylogenetics.

I confirm that I have read this submission and believe that I have an appropriate level of expertise to confirm that it is of an acceptable scientific standard.

# Reviewer report: Reviewer response for version 1

**DOI:** 10.21956/wellcomeopenres.25766.r113689

**Published:** 2024-12-31

**Authors:** Yu-Feng Huang

**Affiliations:** 1 National Chung Hsing University, Taichung, Taiwan

The manuscript, “The genome sequence of the Poplar Grey moth, *Subacronicta megacephala*,” reports the first chromosomal-level genome assembly of *S. megacephala*, as part of the Darwin Tree of Life Project. Derived from a male specimen collected in Wytham Woods, UK, the assembly spans 424.20 Mb, with 99.02% scaffolded into 31 chromosomal pseudomolecules, including the Z sex chromosome. Using PacBio HiFi and Hi-C data, the assembly achieved a scaffold N50 of 14.8 Mb and 97.5% BUSCO completeness. Annotation identified 18,189 protein-coding genes, supported by robust quality metrics, including a QV of 58.2 and 100% k-mer completeness.

 The study highlights the ecological significance of *S. megacephala* and its interactions with Populus species, providing insights into Lepidopteran genetics. Advanced tools like Hifiasm and MERQURY were used for evaluation, and the assembly includes a polished primary haplotype and an unphased mitochondrial genome. However, there are some issues should be addressed.

1. The author should provide a workflow diagram of the bioinformatics pipeline for genome assembly, annotation, and evaluation to enhance clarity.
2. Page 5: DNA concentration was quantified using the Qubit Fluorometer v2.0 and Qubit HS Assay Kit and the final library fragment size analysis was carried out using Agilent Femto Pulse Automated Pulsed Field CE Instrument and gDNA 165kb gDNA and 55kb BAC analysis kit. —> please check “gDNA 165kb gDNA and 55kb”

Are sufficient details of methods and materials provided to allow replication by others?

Yes

Is the rationale for creating the dataset(s) clearly described?

Yes

Are the datasets clearly presented in a useable and accessible format?

Yes

Are the protocols appropriate and is the work technically sound?

Yes

Reviewer Expertise:

Bioinformatics, structural bioinformatics, mitochondrial genome assembly and annotation,

I confirm that I have read this submission and believe that I have an appropriate level of expertise to confirm that it is of an acceptable scientific standard, however I have significant reservations, as outlined above.
